# Resistance and durability of fly ash based geopolymer for heavy metal immobilization: properties and mechanism†

**DOI:** 10.1039/d4ra00617h

**Published:** 2024-04-18

**Authors:** Xupicheng Ren, Fan Wang, Xiang He, Xiaomin Hu

**Affiliations:** a Institute of Resource and Civil Engineering, Northeastern University Shenyang 110014 P.R.China hxmin_jj@163.com +86-2483679128; b Liaoning HaiTianGe Enviromental Protection Technology Co. Ltd Fushun 113122 P.R.China; c Nanning College for Vocational Technology Nanning 530000 P.R.China

## Abstract

Geopolymer technology is an effective method of fly ash (FA) disposal developed in recent decades. This study provided a novel technology based on geopolymerization for FA resource, which could solve the problem of long-term heavy metal leakage trends. Firstly, Unconfine compressive strength (UCS) of geopolymer and the heavy metals leaching test was taken to discuss the effects of oxidize species. The results indicated that the UCS of geopolymer samples was increased with the increase of CaO, and the largest 28 d UCS was 24.8 MPa when CaO content was 31.5%. When the CaO content was 32%, the leaching concentration of heavy metals was the lowest (Pb^2+^ was 0.02 mg L^−1^, Cd^2+^ was 0.01 mg L^−1^), and the solidification rate of heavy metal ions were more than 93.6%. Secondly, two methods were used to evaluate the corrosion resistance of FA based geopolymer. The observations suggested that the FA based geopolymer exhibits a high level of resistance to erosion caused by sulfate ions and chloride ions. Thirdly, carbonation tests were taken to discuss the durability of FA based geopolymer. The results shown that UCS exhibited a modest rise following the process of carbonation, and then demonstrated a stable trend after a period of 28 days, and the heavy metal leaching test results that comply with the limitations specified in the national standard at 7, 14, 28, and 56 days. The findings from accelerated carbonization tests at 56 days, determined by empirical equations, suggest that the carbonization age of geopolymers is projected to be 102 years. Finally, XRD, FTIR and SEM were taken to discuss the microstructure characterization of FA based geopolymer, and solidification mechanisms of heavy metal ions by geopolymer materials could be concluded as gelation, physical encapsulation, and chemical reactions.

## Abstract 2

This study employed geopolymerization technique to solidify MSWI fly ash, aiming to facilitate its reutilization. The FA based geopolymers exhibit strong durability and mechanical qualities, making them suitable for a variety of applications.

## Graphical abstract

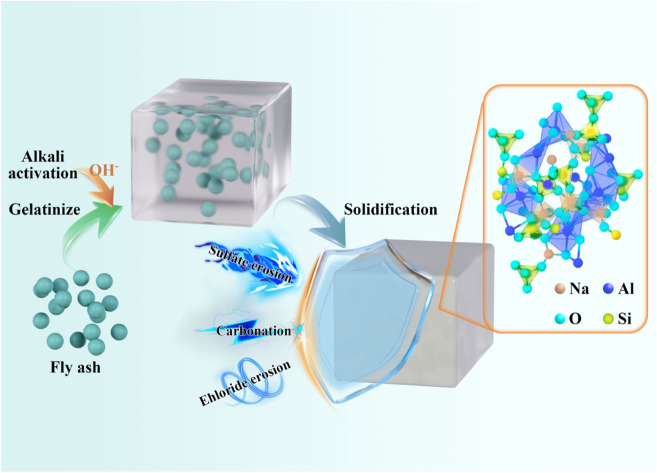

## 1. Introduction

Incineration is a prevalent technique for managing municipal solid waste in China. The process of treating fly ash resulting from incineration is frequently hindered by concerns regarding its efficiency and cost-effectiveness in solidification.^1–3^ It is noted for its effectiveness in completely destroying harmful microorganisms and reducing the volume and weight of MSW by more than 85%. China's estimated municipal solid waste incineration (MSWI) capacity is expected to reach approximately 200 million tons, which is equivalent to 600 000 tons per d, from 2021 to 2025. This facility is projected to produce 6 million tons of MSWI fly ash (FA) annually, with a production rate of 3%.^4^ China's hazardous waste management is greatly concerned about the prevalence of carcinogenic and highly toxic dioxins, heavy metals, and other pollutants in FA.^3^ The emissions from China's MSWI plants are widely recognized as the main contributors to pollution. Consequently, the proper disposal of FA has become a significant concern within the domestic sector. The essential aspect of identifying an economically feasible, secure, and effective approach for disposing of FA is a primary problem.

The main treatment methods for FA now include safe disposal in landfills, solidification/stabilization (S/S), separation and extraction, and heat treatment.^5^ Out of the various methods used in China for disposing of FA, the S/S technology is considered the most prominent.^6^ S/S is a procedure that entails the amalgamation of a fixing agent with an immobilization agent, followed by a reaction conducted under precise conditions to generate a solidified matrix. The stabilization of FA primarily takes place through physicochemical processes, leading to the creation of a stable compound that has minimal toxicity and is highly resistant to migration or dissolution.

Geopolymer technology is an effective method of FA disposal developed in recent decades.^7–9^ The process of FA-based geopolymer curing stabilization involves mixing FA with a geopolymer matrix and adding alkali exciters. This mixture undergoes a geopolymerization reaction, resulting in the formation of a geopolymer that stabilizes the FA.^10,11^ The geopolymer effectively immobilizes pollutants such as heavy metals and dioxins, as confirmed by leaching tests that comply with national standards. This process allows for the resource utilization of FA according to specific requirements.^12,13^

The stability of FA-based geopolymer in the environment is of great significance, and the degree of influence of the curing body on the natural environment needs to be further confirmed. Sulfate erosion, as the most common type of erosion, should be fully considered before the geopolymer is applied. Sulfate erosion mainly occurs in coastal and inland salt lake areas, especially in the environment of acidic groundwater or high viscous soil, which will have a certain impact on the strength of the geo-polymerization material.^14^ The resistance to chloride ion permeability is also a significant indicator of the erosion resistance of the geopolymer. It measures the extent of diffusion or migration of ions within the structure when exposed to pressure or electric field force. This resistance is influenced by the compactness and pore structure of the cured material.^15^ Furthermore, carbonation test technique and standard of cement (GB/T50082-2009) concrete can be utilized to evaluate the impact of carbonation on the FA based geopolymer. The carbonation depth of concrete specimens in a specific concentration of CO_2_ medium can be used to evaluate the long-term stability (or durability) of the concrete.^16^ Zhang^17^ discovered a relationship between natural carbonation and artificially accelerated carbonation. This correlation formula may be utilized to calculate carbonation rate coefficients and anticipate the carbonation process. This study introduced a novel approach utilizing geopolymerization reaction to treat MSWI FA. This method addressed the issue of FA disposal and transformed it into a valuable resource. Furthermore, geopolymers offered benefits such as high early strength, low exothermic reactions, durability, and long-lasting curing compared to Portland cement. Additionally, the preparation of geopolymers eliminated the need for milling and calcination processes, resulting in carbon dioxide emissions reduction.^6^

The primary research objective of this work is: 1, to examine effect of oxidize species on the UCS of FA-based geopolymer; 2, to explore the effectiveness of the FA-based geopolymer immobilization of curing heavy metals; 3, to investigate the FA-base geopolymer sulfate resistance and resistance to chlorine ion penetration performance; 4, to carry out the geopolymer carbonization and to predict the long-term stability of the FA based geopolymer through a comprehensive comparison; 5, to detect the geopolymer microscopic structure and the curing mechanism.

## 2. Materials and methods

### 2.1 Solid materials

The primary compositions of slag and metakaolin chosen for this investigation could be found in Table S7,† and their micro-morphology was displayed in Fig. S3.† Slag complies with GB/T 25 032-2010 standard for slag aggregate used in MSWI and could be used directly as an aggregate for cement products, demonstrating its environmental friendliness.

The geopolymerization processes primarily depend on the dissolution and gelation of silica–aluminate in an alkaline setting. Therefore, the FA utilized in this study should be batches rich in silica–aluminate. Metakaolin and slag were added to MSWI FA to ensure there was sufficient silica–aluminate content for a sufficient reaction, as the source of silica–aluminate.

This project created a geopolymer using hazardous waste FA as a raw material. The MSWI FA was sourced from Everbright Environmental Protection Energy (Shenyang) Co., Ltd, and the samples were collected from the bag-type dust collector within the MSWI system. The chemical analysis of FA was presented in Tables S1 and S2.†The pre-treatment method is to dry for 12 h in a 108 °C dryer.

The slag and metakaolin, utilized as the raw materials for have plenty Si and Al elements good for geo-polyamidation in this study, were sourced from the Qingling Mineral Processing Plant located in Hebei Province, China. Metakaolin is derived through the process of calcination, wherein kaolin (AlO·2SiO_2_·2H_2_O) was subjected to elevated temperatures ranging from 600 °C to 900 °C for a duration of 2 h.

In this investigation, slag was included to adjust the silica–aluminate ratio, whereas FA served as both a fixed component and actively participated in the reaction. This is an important innovation compared with previous studies.^18^

### 2.2 Liquid materials

The excitation curing liquid utilized in this study comprises of water glass, specifically sodium silicate (Na_2_O·*n*SiO_2_), along with solutions of aluminum silicate (NaAlO_2_), aluminum hydroxide (Al(OH)_3_), and sodium hydroxide (NaOH). The refractive index of water glass was determined by the ratio of SiO_2_/Na_2_O, ranging from 1.5 to 3.5. In order to achieve the desired refractive index, NaOH was commonly employed for adjustment purposes. All the liquid components mentioned are of analytical quality and were bought from Sinopharm Chemical Reagent Co. Ltd. The water glass criterion is 25 kilograms per barrel of aqueous solution, whereas the Al(OH)_3_ and NaOH solution are prepared from 500 grams each bottle of solid reagent.

### 2.3 Synthesis of FA based geopolymer

A diagram of the FA based geopolymer has been synthesis in Fig. S1.† The preparation processes consist of the subsequent steps: Firstly, the experimental procedures involved measuring a specific quantity of FA and a mineral regulator mixture, consisting of a geo-polymerization curing agent and a geo-polymerization curing liquid, in accordance with a predetermined ratio. The mixture was then placed in a specialized stirrer and stirred for a duration of 90–120 seconds until it achieved a uniform consistency, characterized by the absence of visible grains and a certain level of fluidity. The mixture has been stirred continuously until it reached a specific level of fluidity and viscosity. Subsequently, the mixture was injected into a cement mortar triple test mold with dimensions of 40 mm × 40 mm × 160 mm. The test mold was placed on a vibrating table to facilitate compaction through vibration. Following this, the test mold was left undisturbed in ambient air conditions for 24 h. The manufactured FA-based geopolymer was placed into a curing box that maintains a constant temperature and humidity. The temperature was regulated at 20 ± 2 °C, while the humidity was maintained at a level equal to or more than 95%. Following the designated curing period, the geopolymer was removed from the curing box at the appropriate age.

### 2.4 Unconfined compressive strength (UCS)

The UCS of the geopolymer samples with different curing times and total contents were examined to reveal the effect of curing time and total contents on their mechanical strength. The UCS was determined using an unconfined compression-testing machine (DYE-300, Jinan, China). Each batch was repeated three times and the average values were calculated. The equation utilized for determining the UCS is:1
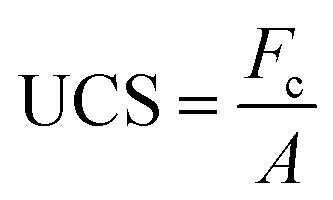
where: UCS—compressive strength, MPa; *F*_c_—maximum load at failure, N; *A*—compression area, mm^2^.

### 2.5 Heavy metal immobilization rate

The geopolymers were tested using the Solid Waste-Leaching Method for Leaching Toxicity-Acetic Acid Buffer Solution Method (HJ/T 300-2007), the Solid Waste-Leaching Method for Leaching Toxicity-Horizontal Oscillation Method (HJ/T 557-2010), and the National Standard Solid Waste-Determination of Copper, Zinc, Lead and Cadmium-Atomic Absorption Spectrophotometric Method (GB/T 15 555.2-1995). Details in Test S1.

### 2.6 Sulfate resistance of the geopolymer

The geopolymer test blocks were partitioned into two distinct groups for experimentation. The first group served as a blank control group and was subjected to immersion in water. The second group, was exposed to a pre-prepared solution consisting of either 5% Na_2_SO_4_ or MgSO_4_. To mitigate water evaporation throughout the soaking procedure, a layer of plastic wrap was applied to the surface of the container, and the solution was refreshed on a weekly basis. Once the test block has been immersed for the designated duration, it should be removed, the surface wiped, and subsequently subjected to quality and UCS testing.

The calculation of the mass loss rate is determined by the following equation:2
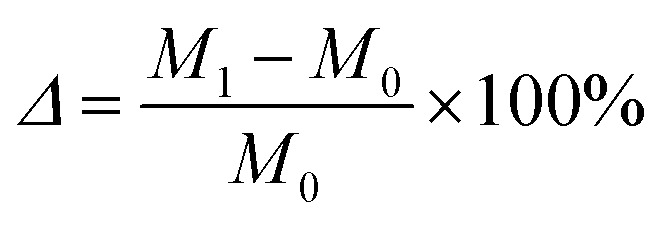
where: *Δ* – mass loss rate, %; *M*_1_-mass of cured body in Na_2_SO_4_ solution or MgSO_4_ solution, accurate to 0.1 g; *M*_0_-mass of cured body in water, accurate to 0.1 g.

The corrosion resistance coefficient is calculated as follows:3
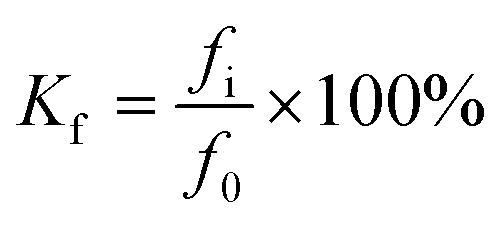
where: *K*_f_-compressive strength corrosion resistance coefficient; *f*_i_ – the UCS of the cured body test block after sulfate erosion, accurate to 0.1 MPa; *f*_0_ – the UCS of the curing body test block, while immersed in water, is measured with an accuracy of 0.1 MPa.

### 2.7 Chloride ion penetration test

The examinations were carried out in compliance with the Standard for Test Methods of Long-term Performance and Durability of Ordinary Concrete (GB/T 50 082-2009). Details in Test S2.

### 2.8 Carbonization experiment

The test processes were as follows: FA-based geopolymers were prepared by adding inorganic curing agent at 25% of water consumption, and standard curings were carried out under the same conditions. After standard curings for 28 days, the FA was dried at 60 °C for 48 h, and the FA solidified bodies were filled into a transparent plastic beaker according to the actual bulk density when stored in a ton bag. Sixteen samples were prepared (4 for phenolphthalein titration to confirm the carbonation depth, 4 for water extraction test, 4 for acetic acid extraction test, and 4 for sulfuric acid and nitric acid extraction test). Carbonization chamber test conditions: carbon dioxide concentration 20 ± 3%, temperature 20 ± 5 °C, humidity 70 ± 5%. Details in Test S3.

### 2.9 Characterization of geopolymer

The mineralogical compositions of the geopolymer samples were analyzed using X-ray diffraction (XRD, D8 Advance diffractometer, Germany). The determination of the alteration in binding energy and valence states of elements inside the geopolymers were conducted using Fourier Transform Infrared Spectroscopy (FTIR, Thermo Nicolet instrument, United States). The morphology of the geopolymers were analyzed using scanning electron microscopy-energy dispersive spectroscopy (SEM, Hitachi S4800, Japan).

## 3. Results and discussion

### 3.1 Effect of oxide contents on the UCS of geopolymer

Tables S1 and S2† display the chemical compositions of FA from various regions and batches. The findings from the tables indicated that the FA utilized in this study has a high calcium content and low levels of silica and aluminum. Due to the influence of FA batch, region and mineral regulator type, the oxide composition of FA and mineral regulator from different regions is obviously different, and the oxide composition determines the structure type of the geopolymer. Therefore, in the following study, the influences of the oxide composition in the geopolymer on the mechanical properties of the geopolymer were mainly considered.

#### 3.1.1 FA contents

**Fig. 1:**
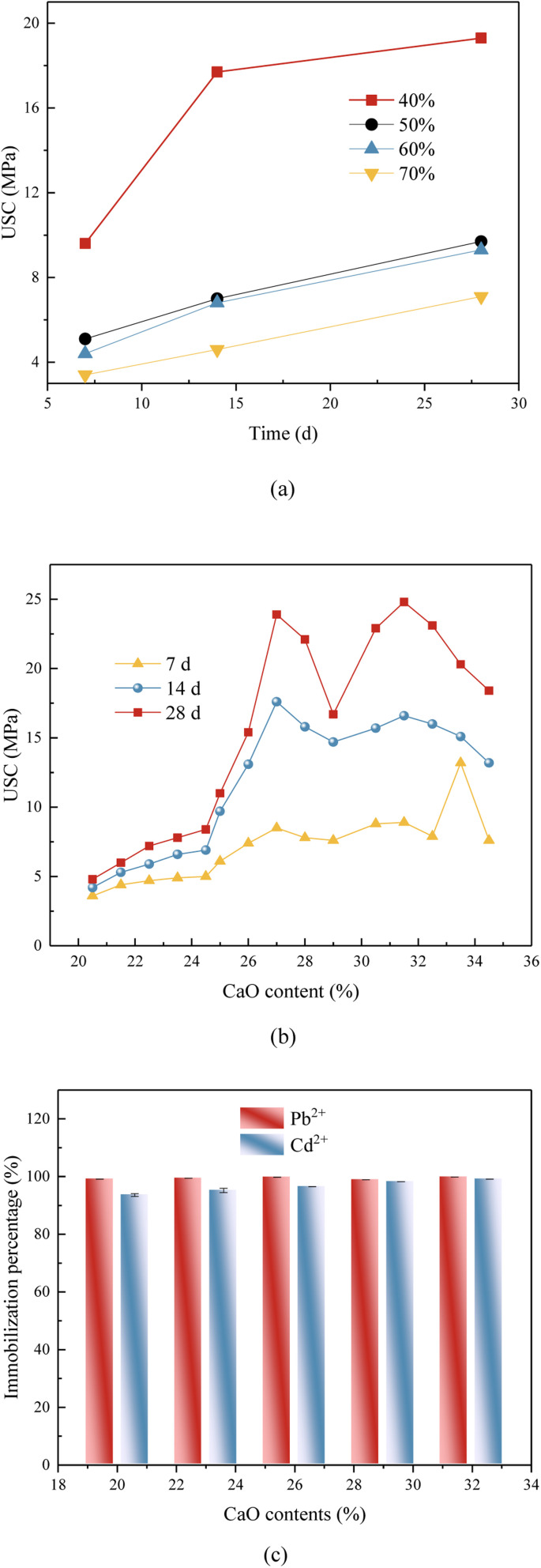
Effect of oxidize species content on UCS of geopolymer and heavy metal immobilization efficiency. (a) Effect of FA contents on UCS; (b) effect of CaO contents on UCS; (c) effect of CaO contents on heavy metal immobilization efficiency.

The proportion of Ca in the FA was excessive, while the quantity of Si and Al crucial for the geo-polymerization process was inadequate. Therefore, the amount of added FA might significantly impact the UCS of FA-based geopolymer. The UCS of geopolymer for different FA contents was shown in Fig. 1a, the 7 d, 14 d and 28 d UCS of the geopolymer gradually decreased with the increase of FA content. When the FA content was 50%, the 28 d UCS of the geopolymer was the highest, which was 19.3 MPa. When the FA content was 80%, the 28 d UCS was the lowest 7.1 MPa. A higher FA content leaded to a decrease in the amount of mineral modifier, and a reduction in Si–Al content in the system, which restricted the creation of the geopolymeric structure. With the increase of FA content, the restriction effect was more obvious, which also leaded to a steady decrease in the UCS of the geopolymer. Based on this, in the follow study, the FA content of 50% would be selected to investigate the parameters that influence the UCS of the geopolymer.

#### 3.1.2 CaO contents

Previous researches has demonstrated that the Ca content could influence the course of the geo-polymerization processes then significantly affecting the UCS of geopolymers.^19^ The effect of CaO contents on UCS of FA-based geopolymer was explored. CaO was mainly provided by FA and slag, and the content of CaO could be changed by adjusting the content of slag when the content of FA was 50%. As depicted in Fig. 1b, content of CaO was changed from 20.5% to 34.5%, with the increase of CaO content in the geopolymer, the UCS of the geopolymer at 7 d, 14 d and 28 d increased first and then decreased. The UCS at 28 d was only 4.8 MPa when the CaO content was 20.5%. The 28 d UCS reached its maximum value of 24.8 MPa when the CaO concentration was around 31.5%.

There might be three reasons for this phenomenon: (i) dense structures were formed. CaO would react with H_2_O in the alkali activator to form Ca(OH)_2_, and then Ca(OH)_2_ reacted with CO_2_ in the air to form dense mineral systems such as CaCO_3_.^20,21^ At the same time, the generated Ca(OH)_2_ was alkaline in water, which provided conditions for the active reaction of metakaolin.^22^ (ii) The Si–Al chain structure became more stable and exhibited better compressive strength. With the increase of calcium content in the system, the average bond length of the Ca–O bond became shorter, indicated that the increase of calcium content would promote the bonding of Ca–O, which made the average bond length of the Si–O bond in the structure shorter, and the Si–Al chain structure became more stable and exhibited better compressive strength. (iii) More stronger chain types were formed. With the increase of CaO content, the content of Si–Al (mainly SiO_2_ and Al_2_O_3_) in the system was also increasing. According to the research of Tian *et al.*,^23^ the unit structure of geopolymer could be divided into PSDS type (–Si–O–Al–O–Si–O–Si–O–), PSS type (–Si–O–Al–O–Si–O–) and PS type (–Si–O–Al–O–), of which PSS type was the long chain structure of Si and Al was the strongest chain type. CaO, SiO_2_ and Al_2_O_3_ in the raw materials reacted to form C–S–H and C–(A)–S–H gel structures under the condition of alkali activation, which were attached to the three-dimensional structure of zeolites generated by the previous polymerization reaction, which was equivalent to filling the gaps and making the structure more compact, thus improving the UCS.^24^

When CaO content was 34.5% the 28 d-UCS was dropped to 18.4 MPa. Since the content of FA was constant, the higher of content of CaO the more slag was added to the raw material. Due to the increase of slag amount, the increase of C–(A)–S–H in the system might cause certain damage to the grid structure, which made the UCS of the geopolymer start to decline in the macro level. On the other hand, with the increasing of CaO content, the unit structure of geopolymer begin to transform from PS-type and PSS-type to PSDS-type gradually, and the PSDS-type geopolymer structure has some side effects on SiO_4_, which reduces the structural stability.

#### 3.1.3 Ratio of SiO_2_/Al_2_O_3_

The main component of the geopolymer is an oxide combination including Si and Al. The geopolymer's three-dimensional structure resembles that of zeolite, and it is comprised of SiO_4_ and AlO_4_ units.^25^ Hence, Si and Al assumed a significant role in the development of the geopolymer structure. The SiO_2_/Al_2_O_3_ ratio might significantly affect the UCS of the geopolymer.

In the high calcium system, the molar ratio of *n* (SiO_2_)/*n* (Al_2_O_3_) ranged from 4.5 to 4.9. The UCS of the geopolymer, depicted in the Table 1, at 7 days, 14 days, and 28 days exhibited a statistically significant increase with the increase of *n* (SiO_2_)/*n* (Al_2_O_3_). At a molar ratio of *n* (SiO_2_)/*n* (Al_2_O_3_) of 4.7, the UCS of the geopolymer reached its maximum value of 15.2 MPa after 28 days. In the high calcium system, the geopolymer exhibited a significant slag content and contained a substantial quantity of CaO. The CaO component underwent a reaction with SiO_2_ and Al_2_O_3_, resulting in the formation of a C–(A)–S–H gel that possessed a specific level of strength.^26^ Nevertheless, as the molar ratio of *n* (SiO_2_)/*n* (Al_2_O_3_) increased, the concentration of Al_2_O_3_ in the system diminished, leading to a reduction in –Si–O–Al chains. Consequently, a portion of SiO_2_ remains unreacted, resulting in a decline in the geopolymer's strength.

**Table 1:** Effect of *n* (SiO_2_)/*n* (Al_2_O_3_) ratio on UCS of FA based geopolymer

*n* (SiO_2_)/*n* (Al_2_O_3_)	7 d	14 d	28 d
4.5	8.9	9.2	11.1
4.6	9.1	11.7	12.9
4.7	11.7	14.1	15.2
4.8	10.4	12.9	13.3
4.9	8.8	10.0	11.7

### 3.2 Immobilization efficiency of heavy metal

Fig. 1c revealed that the immobilization efficacy of heavy metal in the geopolymer solidified during geo-polymerization was much inferior to that observed in the original FA (Tables S3 and S4†). The immobilization efficiency of heavy metals in the geopolymer exhibited a progressive increase with the rise in CaO content. The geopolymer exhibited the highest immobilization efficiency for heavy metals when the CaO content was 32%. At this composition, the leaching concentrations of Pb^2+^ and Cd^2+^ were measured to be 0.02 mg L^−1^ and 0.01 mg L^−1^, respectively. Furthermore, the maximum amounts of Cd and Pb that could be trapped per gram of geopolymer were determined to be 0.191 mg g^−1^ and 0.047 mg g^−1^, respectively. Following the process of geo-polymerization, the S/S efficiency for Pb^2+^ and Cd^2+^ could attain a notable rate of 93.6%. The favorable solidification phenomenon observed in geopolymer materials might be attributed to the involvement of heavy metal present in FA during the three-dimensional structure creation reaction of the geopolymer. Table 4 presented the immobilization efficiencies of some commonly used methods. Cement curing is costly and can be adjusted in size, but there is a risk of leakage. Chemical stabilization is cheap but needs a landfill site. Melt curing is expensive and consumes a lot of energy. The adsorption method is restricted by the adsorption material and lacks long-term stability. In conclusion, geopolymerization technology offers specific benefits.

### 3.3 Erosion resistance experiments

#### 3.3.1 Study on sulfate erosion resistance of geopolymers

The assessment of the geopolymer's resistance to sulfate was conducted by analyzing its mass loss rate and corrosion resistance coefficient (*K*_t_). According to the observations depicted in Fig. S1,† there was no significant detachment or porous structure on the surface of the all three group of geopolymers. Table 2 displayed the observed alterations in the mass of geopolymers following exposure to sulfate erosion for several days.

**Table 2:** Variation in mass and UCS of FA based geopolymer following sulfate attack

CaO content		7 d	14 d	28 d
31.5%	Mass in pure water (g)			
	Mass in Na_2_SO_4_ aq. (g)	421.2	421.7	421.6
	Mass in MgSO_4_ aq. (g)	423.2	423.0	423.4
	Mass loss rate in Na_2_SO_4_ aq. (%)	0.00	0.31	0.50
	Mass loss rate in MgSO_4_ aq. (%)	0.64	0.62	0.93
	UCS in pure water (MPa)	12.3	13.5	17.6
	UCS in Na_2_SO_4_ aq. (MPa)	13.9	14.4	16.7
	UCS in Na_2_SO_4_ aq. (MPa)	12.0	11.3	16.2
	*K*_t_ in Na_2_SO_4_ aq.	1.13	1.07	0.95
	*K*_t_ in MgSO_4_ aq.	0.98	0.84	0.92

Based on Table 2, the quality of the cured body exhibited a marginal decline as the duration of immersion in clear water increased. The slight augmentation in the mass of the solidified specimen upon immersion in a solution of MgSO_4_ could potentially be attributed to two factors. Firstly, it was possible that some of the MgSO_4_ crystals adhered to the surface of the geopolymer, thereby leading to an increase in mass. Secondly, it was also possible that the constituents present in the FA underwent a chemical reaction with the MgSO_4_, resulting in the formation of chemical byproducts and subsequently contributing to the observed mass increase. The mass loss rate of the geopolymer in Na_2_SO_4_ solution exhibits a notable increase, primarily attributed to the formation of the N–A–S–H gel structure. This gel structure demonstrated a high level of resistance against sulfate erosion.^27,28^

Table 2 present the UCS and *K*_t_ of the geopolymer specimens following immersion in sulfate solution for durations of 7, 14, and 28 days. The UCS of the geopolymer specimens exhibited an upward trend as the immersion time in a Na_2_SO_4_ solution with a mass fraction of 5% increased. Conversely, in the case of immersion in a MgSO_4_ solution, the UCS initially decreased and then increased with the increase in soaking time. This phenomenon might be attribute to Na_2_SO_4_ solution provided plenty of Na^+^, and the period of N–A–S–H gel was a longtime reaction, so the formation of N–A–S–H provide a growing UCS.^19^ When examining the UCS of the geopolymer submerged in water and comparing it to the applicable national standards for concrete durability, it became evident that the *K*_t_ of the geopolymer in sulfate solution was greater than 0.8. These findings served as evidences that the FA-based geopolymer exhibited favorable resistance to sulfate erosion.

#### 3.3.2 Study on resistance to chloride ion erosion of geopolymers

**Fig. 2:**
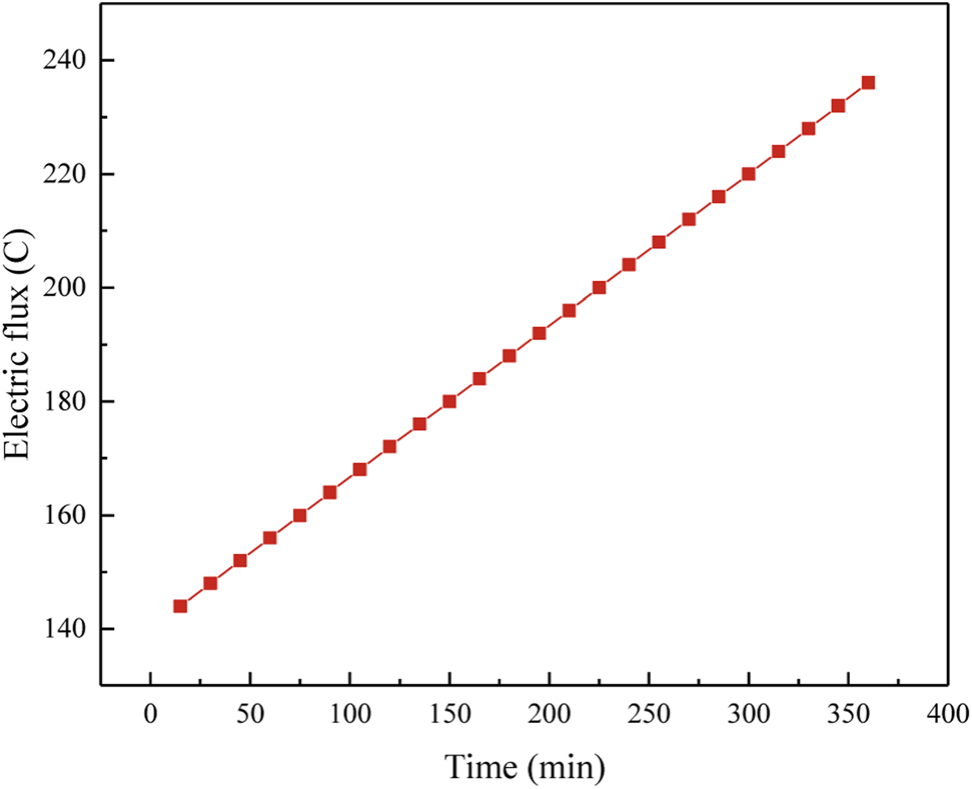
Electric flux values changed with time of power-on time.

Table S5† presented the assessment range of resistance to chloride ion permeability and its correlation with the permeability of the geopolymer. Fig. 2 illustrated the impact of the energization duration of chloride ions on the energization quantity of the geopolymer within the high-calcium systems (CaO content higher than 30%). As the duration of energization raised, the amount of energization in the geopolymer also grown linearly. However, the cumulative energization amount remained below 250 °C within a 6 hour period. The presence of a significant amount of CaO in the system leaded to a substantial occurrence of the polymerization reaction, resulting in a greater stability of the three-dimensional skeleton. Consequently, the internal pores were diminished, causing water molecules to migrate towards the internal porous space, thereby enhancing their stability. The reduction in internal pore space leaded to a decrease in the movement of water molecules, resulting in a relatively low macroscopic flux performance.

This observation suggested that the FA-based geopolymer exhibited a high level of resistance to erosion caused by chloride ions. There might be two reasons for high resistance of FA based-geopolymer: (i) the FA-based geopolymer matrix has a low porosity and a compact structure, creating a barrier on the surface that prevents sulfate and chloride ions from penetrating into the inside, thus inhibiting erosion; (ii) the micro-structure produced during geo-polymerization have a more stable three-dimensional configuration, making them less susceptible to chemical reactions and enhancing their resistance to erosion.

### 3.4 Carbonization test and durability life research

#### 3.4.1 UCS of geopolymers before and after carbonation

**Fig. 3:**
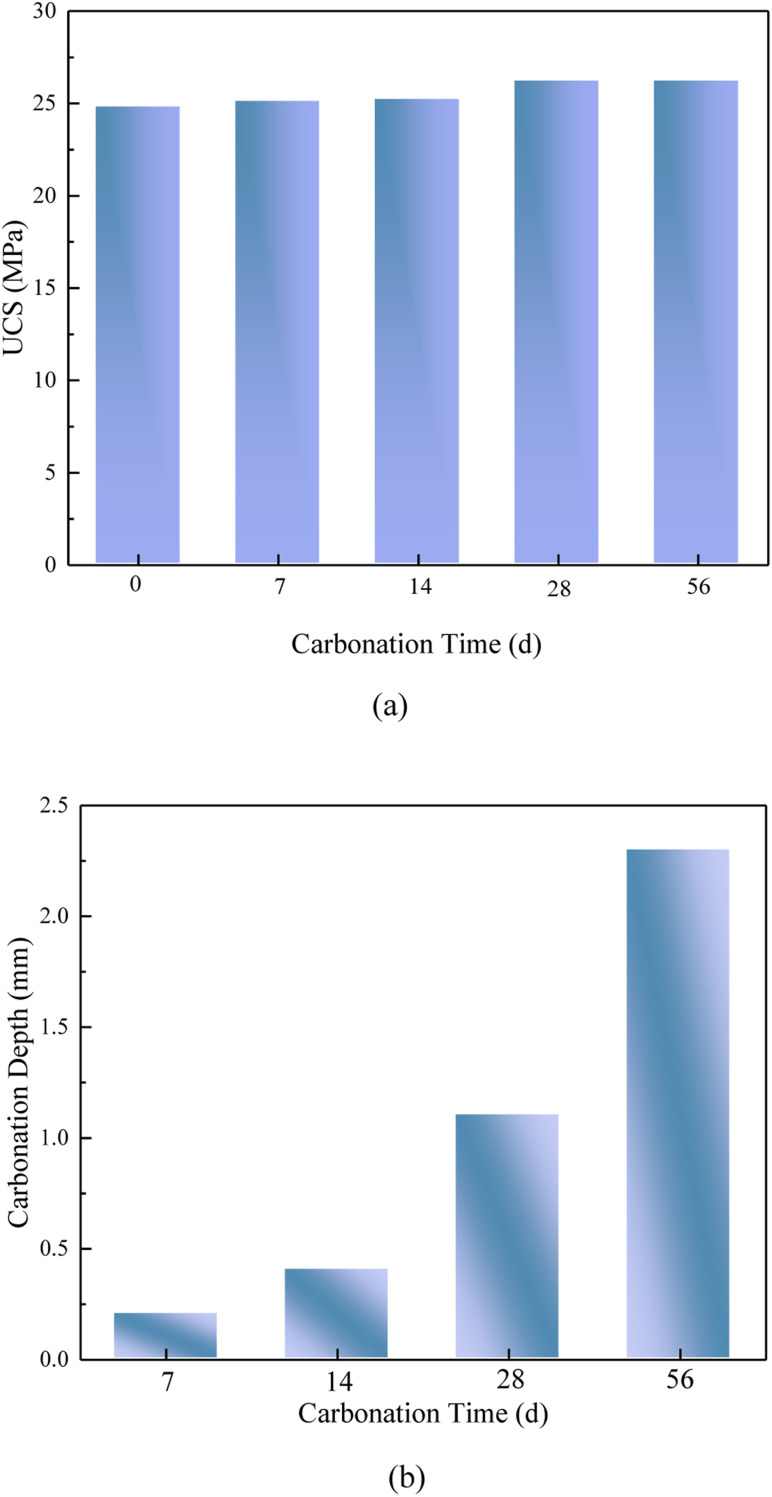
UCS and carbonation depth of FA based geopolymer. (a) UCS; (b) carbonation depth.

The UCS of FA-based geopolymer following accelerated carbonation for 7, 14, 28, and 56 days could be observed in Fig. 3a. The corresponding values were 9 to 14 MPa, 10 to 14 MPa, 10 to 16 MPa, and 10 to 16 MPa, respectively. The UCS exhibited a modest rise following the process of carbonation, and then demonstrated a stable trend after a period of 28 days.

#### 3.4.2 Heavy metal leaching analysis before and after carbonization

The leaching results of heavy metals from FA-based geopolymer samples, have undergone 28 days of maintenance, were presented in Table 3. According to the data presented in Table 3, the levels of Cd and Pb in the FA were found to above the threshold established by the GB 16889-2008 standard by a factor exceeding 20. Upon undergoing geo-polymerization, the concentration of heavy metal leaching in samples complied with the prescribed limitations set by the national standard. After 7 d, 14 d, 28 d and 56 d accelerated carbonization of FA-based geopolymer, the heavy metal leaching test met the requirements of national standard limits.

**Table 3:** Heavy metal concentration of FA and FA based geopolymer leaching test

	Cd	Pb	As	Ba	Be	Cr	Cu	Hg	Ni	Se	Zn
Standard	0.15	0.25	0.3	25	0.02	4.5	40	0.05	0.5	0.1	100
FA	4.236	4.771	0.079	3.313	0	0.072	2.385	0.063	0	0.005	28.28
0 d	0	0.071	0.005	1.506	0	0	0	0	0.046	0.040	0.171
7 d	0.005	0.043	0	1.258	0	0.021	0.037	0	0.088	0.036	0.123
14 d	0.005	0.086	0	1.282	0.001	0.030	0.042	0	0.072	0.043	0.165
28 d	0.007	0.067	0	1.351	0	0.022	0.042	0	0.083	0.033	0.144
56 d	0.002	0.051	0	1.221	0	0.018	0.039	0	0.090	0.042	0.139

**Table 4:** Presentation of other heavy metal immobilization methods

Curing method for heavy metal ions	Cement curing method^34^	Chemical stabilization^57^	Melt-curing method^58^	Adsorption^59^
Advantages	Economical and easy to operate	Small volume increment, reagents can be selected according to heavy metal species	Low volume increase rate, good curing effect	Raw materials are easily available and the process is simple
Disadvantages	Pb^2+^, Zn^2+^ works well, Cd^2+^*etc.* not so well	Complex process, expensive chemicals, possible by-products	Complex process, high operating costs, poor economy	Huge equipment, by-products, easy to cause secondary pollution
Fixation effect	Fixation rate greater than 98% for effective ions	Generally greater than 98%	No heavy metal ions detected in the leaching solution	Typically greater than 90% fixation rate
cost (*R*_mb_/*t*)	1000–2000	600–800	2000–3000	Depends on the market price of the adsorbent material
Scalability	Can be used as a building material within certain limits	There are currently no extended applications	There are currently no extended applications	There are currently no extended applications
Environmental impact	The manufacture of cement products is not conducive to reducing carbon emissions	Requires site landfill	Higher energy consumption and more CO_2_ emissions	May require further processing
Long-term stability	Some risk of leakage	Preferably	Preferably	Adsorption is competitive and less stable over time

#### 3.4.3 Carbonization depth

The carbonation process of the geopolymer is similar to the process of the carbonation of the concrete. The FA based geopolymer has a zeolite-like three-dimensional cage structure with porous characteristics. Due to the large amount of unreacted alkaline solution in the pores, it would react with CO_2_ and water in the natural environment to generate bicarbonate, carbonate and other substances, and the carbonized part of the surface of the geopolymer became loose, which leaded to the deepening of carbonation, and the depth of carbonation was gradually increased with the prolongation of time. From Fig. 3b, it could be seen that after 56 d accelerated carbonation of FA based geopolymer, the average depth of carbonation was 2.3 mm, while the accelerated carbonation depth of general concrete could reach a maximum of 20–30 mm, which was 10–15 times higher than that of geopolymer, indicating that the degree of densification of the geopolymer was greater than that of ordinary concrete, and the carbonation resistance was also stronger.

#### 3.4.4 Calculation of carbonation age

In the context of a 56 d accelerated carbonation test using FA-based geopolymer, it is generally accepted that the concentration of CO_2_ in the atmosphere can be considered as 0.03%. By employing the carbonation depth formula (eqn (S2)†), it was evident that for a given carbonation depth, the accelerated carbonation age and the natural carbonation age exhibit a correspondence, as presented in Table S6.† Specifically, the accelerated carbonation ages of 7, 14, 28, and 56 days correspond to natural carbonation ages of 13, 26, 51, and 102 years, respectively.

Table S8† displayed the leaching of heavy metals following the carbonization of the geopolymerized curing body, allowing for a comparison with the threshold values outlined in several Chinese regulations. The heavy metal leaching concentration after carbonization remains 2–3 times below the norm. This complies with the criteria for ordinary industrial solid waste. The leached pollutant concentrations of the solidified body meet the soil, groundwater and surface water standards for heavy metals and dioxins, ensuring ecological safety in the environment. The curing body is likely to ensure a safe level of pollutant leaching over time. It is mainly used inside domestic landfills and incineration plants to allow *in situ* resource utilization of MSWI FA.

### 3.5 Characterization of FA based geopolymer

#### 3.5.1 XRD analysis

**Fig. 4:**
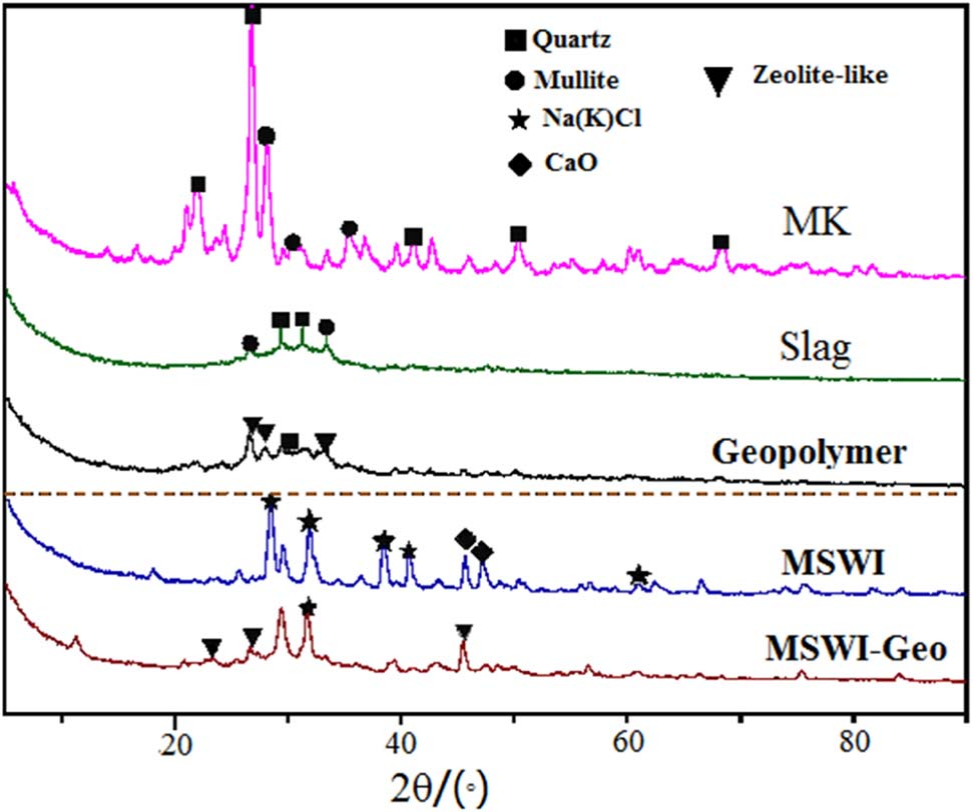
XRD images of raw materials and FA based geopolymer.

Fig. 4 displayed the XRD patterns of the raw materials. The X-ray energy spectrum of the metakaolin sample exhibited a distinct and well-defined crystallization peak within the angular range of 20–30°. This peak has been identified as the X-ray diffraction peak corresponding to the presence of quartz crystal (SiO_2_) and mullite (Al_2_O_3_), as validated by comparison with the standard X-ray film.^29^ The X-ray spectrum of the original FA primarily consists of calcium salt and chlorine salt phases. Additionally, there were minor peaks observed between 50° and 60°, suggesting the presence of a small quantity of quartz phase in the original FA. This observation confirms that the FA exhibited some degree of gelling activity when subjected to alkali excitation.

The composition of slag was intricate; nonetheless, analysis of its X-ray spectra revealed that its predominant phase closely resembles metakaolin, exhibited a peak within the range of 26° to 33°. Furthermore, the slag contained crystalline structures such as quartz (SiO_2_) and mullite (Al_2_O_3_). In the absence of FA addition, the X-ray spectrum of geopolymer exhibited a higher density of the geopolymer gel peak within the angular range of 25° to 35°. The hydrated calcium aluminosilicate peaks were observed at 26.7° and 45.6° in the geopolymer sample, suggesting the presence of heavy metals and contaminants from the FA used in the geopolymer mixture. The presence of interference substances hindered the rate of polymerization reaction. The geopolymer made from FA had a greater CaO content than the pure geopolymer. At a peak of 29.2°, the hydrated calcium silicate structure became evident. This observation suggested that the calcium salt, which was more abundant in the FA, reacted with the active silica present in the raw material under alkaline conditions, resulting in the formation of calcium silicate gel. Comparing the plots of the geopolymer with the fly ash curing body reveals the formation of a new zeolite phase around 46.7°, which aligns closely with the addition of Cao. This outcome thoroughly clarifies the high calcium content, which enhances the mechanical characteristics and curing outcomes of the cured body.^30^

Through a comparison of the X-ray atlas of the geopolymer and the original FA, it was evident that the diffraction peak of the FA within the range of 30° to 50° diminished or became inconspicuous following solidification. Notably, the characteristic diffraction peak of numerous chlorine salt crystals at 30° and 40° emerges in the X-ray atlas of the geopolymer after the FA underwent the reaction. This observation suggests that: (i) FA is not only a waste material but an activator involved in the reaction during the geopolymerization process. (ii) Some dangerous chemicals in FA are integrated into the polymer structure as crystals.^31^

#### 3.5.2 FTIR analysis

**Fig. 5:**
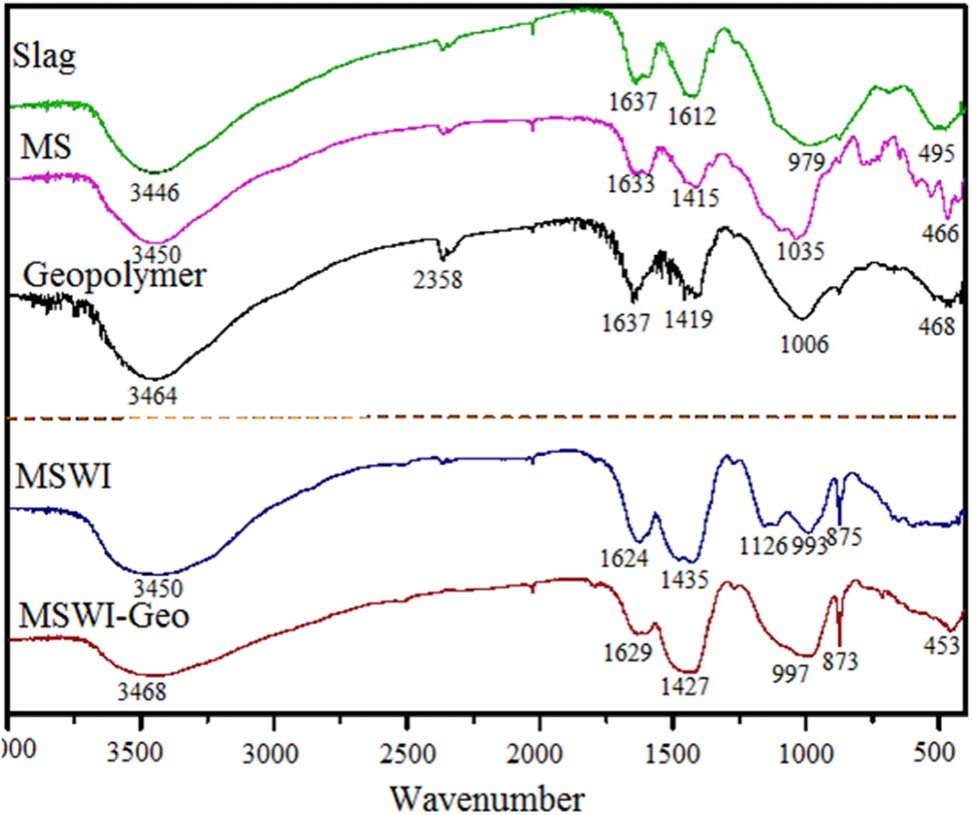
FTIR images of raw materials and FA based geopolymer.

To examine the alterations in the microstructure of both the geopolymer and its constituent raw materials, an infrared spectrum analysis was conducted on the raw materials, pure geopolymer, and FA -based geopolymer utilized in the experiment. The outcomes of this analysis were illustrated in Fig. 5.

The visual evidence presented in the photographs illustrated the process of geopolymer structure creation. A discernible absorption peak of considerable breadth manifested within the wavelength range of 3440–3450 cm^−1^ and at 3450 cm^−1^, signifying the hydroxyl (–OH) stretching vibration of unbound aqueous water. In close proximity, a further peak denotes the OH bending vibration of unbound aqueous water. This suggested that both the raw materials and the geopolymer possess a limited quantity of liquid water. However, the amount of liquid water present was not substantial.^32^ The existence of liquid water within the geopolymer promoted the formation of a denser structure. The absorption peak observed in close proximity signifies the asymmetric stretching vibration of the carbonate ion (CO_3_^2−^) within the CaCO_3_ compound. The infrared spectrum of kaolin revealed that the absorption peak at approximately 1049 cm^−1^ corresponded to the stretching vibration peak of Si–O–A (where A represented Si or Al). Additionally, the absorption peak at around 773 cm^−1^ corresponded to the symmetric vibration peak of Si–O–A, while the absorption peak near 466 cm^−1^ corresponded to the bending vibration peak. In the context of geopolymer research, it has been observed that the bending vibration peak of Si–O–Si or O–Si–O occurs at a wavenumber of 468 cm^−1^ in pure geopolymer. Additionally, the stretching vibration peak of Si–O-A has been identified at a wavenumber of 1006 cm^−1^. The geopolymer exhibits an absorption peak within the range of 986–991 cm^−1^, which corresponded to the stretching vibration peak of Si–O–Al or Si–O–Si. Additionally, another absorption peak was observed at 873–875 cm^−1^, which could be attributed to the symmetric vibration peak of Si–O–Al or Si–O–Si. This microscopic creation elucidates the more rational Al–Si ratio in the fly ash base in contrast to the pure cured body, since the inclusion of calcium content leads to reduced Si–O–Al bond lengths and a more stable structure. This explained how interatomic interactions impact the macroscopic mechanical characteristics and curing process. The absorption peak in 449–455 cm^−1^ corresponded to the bending vibration peak associated with the Si–O–Si or O–Si–O molecular bonds.^33^

Through a comparison of the absorption peak frequencies of metakaolin and the geopolymer, it becomes evident that the stretching vibration peak of Si–O–Si underwent a shift towards lower wavelengths. This shift could be attributed to the stronger bonding force between Si and O compared to Al and O. Consequently, it indicated that the aluminosilicate or silicate structure has undergone a depolymerization reaction.^34^ This suggested that the introduction of Al_2_O_3_ leads to the partial substitution of the group on the Si–O–Si chain in the initial metakaolin.^35^ Additionally, the incorporation of FA allowed for the infiltration of heavy metal ions into the geopolymer structure. Consequently, there was an exchange of vibration energy between the SiO_2_ group and its surrounding environment, resulting in a shift in the position of the stretching vibration peak. In contrast to geopolymer, the stretching vibration peak of Si–O–A in geopolymer exhibited a shift towards lower wavenumbers. This shift suggested a depolymerization of the structure, providing evidence that the utilization of FA as a raw material involved genuine chemical reactions rather than mere physical encapsulation within a three-dimensional cage structure. Simultaneously, it was worth noting that while FA contained a certain quantity of Cl and S, the presence of hydrated calcium chloroaluminate in the resulting product was not seen.^36^ This might be attributed to either the limited quantity of the product or the encapsulation of chlorine and sulfur inside the hydration products formed during the reaction.^37^

From analyzing the infrared spectrum, it can be inferred that: (i) the presence of heavy metals in FA does not affect SiO_4_. The ions were trapped inside a three-dimensional cage-like structure created through geo-polymerization, leading to their solidification. (ii) The macroscopic observation of heavy metal leaching concentration in the geopolymer was conducted. The leaching concentration of heavy metal ions Pb^2+^ and Cd^2+^ in the geopolymer showed a substantial decrease compared to the original FA. (iii) The solidification rate of Pb^2+^ and Cd^2+^ can achieve an astonishing 93.6%. The geopolymeric structure has shown to effectively solidify heavy metals found in FA.

#### 3.5.3 Scanning electron microscope (SEM) analysis

**Fig. 6:**
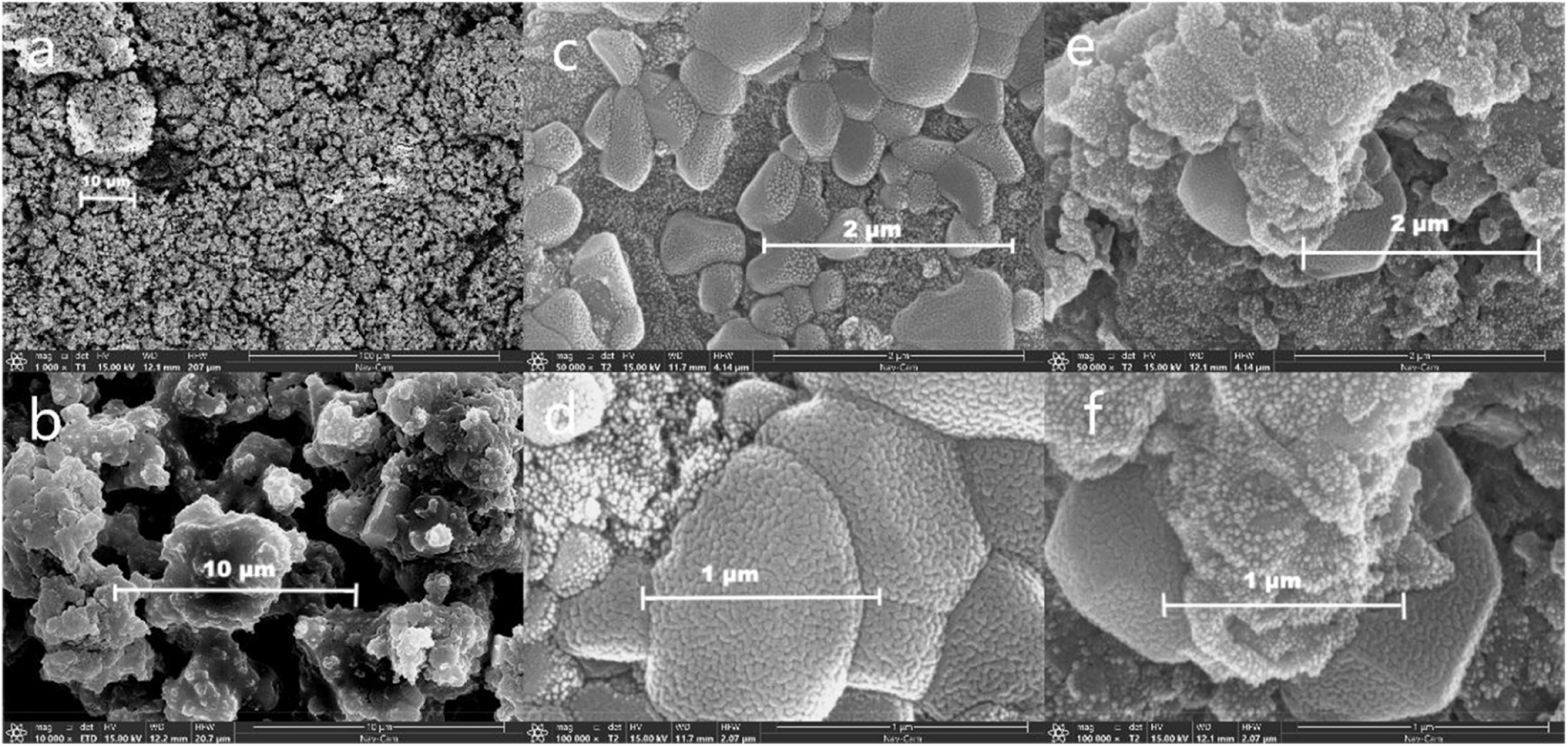
SEM images. (a and b) FA; (c and d) FA based geopolymer before carbonation; (e and f) FA based geopolymer after 28 d carbonation.

Fig. 6 displays the SEM atlas of the geopolymer and raw materials. As evident from Fig. 6a and b, FA consisted of minutely sized gray particles. These particles exhibited a generally loose and irregular distribution. Their shapes were irregular, often flattened, cotton-wool like, flaky, and porous. The particles possessed a rough and reticulated surface, often clustering together. FA predominantly existed in amorphous and polycrystalline forms, with rare instanced of well-defined crystallization. A small number of spherical particles could also be observed, indicated significant differences in the morphology of geopolymerization products.

The analysis of Fig. 6c and d revealed that, a significant portion of the FA actively engaged in the hydration reaction and became integrated into the geopolymer structure. Additionally, a minor fraction of the FA particles adhered to the surface of the geopolymer, likely attributed to the presence of diverse crystal forms within the FA. The irregular crystalline substances were incapable of engaging in the hydration reaction observed in geopolymers. Consequently, these impurities, along with heavy metals, get cemented within the three-dimensional network structure of geopolymers. According to the findings presented in Fig. 6c and d, it was evident that the presence of a high calcium system leaded to a decrease in the aluminosilicate structure, which was primarily composed of the hydration C–A–S–H gel. Instead, the main hydration products in this system shifted towards the hydration C–S–H gel. This gel was characterized by a laminated sheet structure that exhibits strong interconnectivity, which pertained to the immobilization characteristics of geopolymers for heavy metals. The microstructure of FA particles allows heavy metals to be securely embedded in the geopolymer structure. As the reaction progresses, the heavy metal elements become enclosed in the macrostructure. The presence of CaO leads to a compact geopolymer surface with high UCS.^38^

Upon further examination of the geopolymer after accelerated carbonization (Fig. 6e and f), it became evident that the carbonized portion exhibited a more loosely structured particle morphology with increased porosity. This suggested that the carbonization process triggered changes in the geopolymer's internal structure, possibly due to the rearrangement or modification of its chemical components. In contrast, the uncarbonized portion maintained a highly dense internal structure, indicated that it remained relatively unaffected by the carbonization process.

#### 3.5.4 Immobilization mechanism of geopolymer

The reaction between Si and Al in FA and alkali activator results in the development of a polymer mechanism, which is divided into the following steps: 1. During the dissolution step, an alkaline activator dissolves the silicate and aluminate compounds in the FA, creating monomers of silicate and aluminate. 2. Polymerization stage: Monomers undergo a polymerization event to produce dimers, trimers, and other polymers. The alkaline activator serves as a catalyst in the polymerization procedure. 3. Precipitation occurs when the solution becomes saturated, causing the polymer to form a gel. Initially, the gel generated is an aluminum-rich substable gel. During the reaction, the silicate in the FA slowly dissolves, leading to a rise in the silicon content of the gel. A gel phase was eventually generated from the FA-based polymer mass containing C–A–S–H (sodium-containing) and (N,C)–A–S–H (high calcium content N–A–S–H) gels. Hydrated silicates and zeolites are formed as precipitates. Polymerization reaction impacts the structure and pore microstructure of the polymer, therefore influencing the material's physical properties.^39–43^

The solidification mechanisms of heavy metal by geopolymer materials primarily encompass gelation, physical encapsulation, and chemical reactions.^44,45^

Geopolymer compounds solidify heavy metals by gelation, physical encapsulation, and chemical reactions. (i) When exposed to alkali, the amorphous aluminosilicate phase dissolves, resulting in the separation of SiO_4_ and AlO_4_. Afterward, these distinct species experience dehydration polycondensation and gelation processes. (ii) An rise in the concentration of oligomeric gel was noticed as the reaction progressed. This rise resulted in the solidification of heavy metal cations within the system structure, aided by the encapsulation effect. The intended solidification effect was successfully accomplished as a result. (iii)The geopolymer's microporous structure and significant specific surface area help in effectively absorbing heavy metal ions during solidifying. Alkaline circumstances were predominant in this investigation, thus we regard (i) and (ii) as the primary processes by which geopolymers immobilize heavy metal ions.

Upon exposure to alkali, the amorphous aluminosilicate phase undergoes dissolution, leading to the separation of SiO_4_ and AlO_4_. Subsequently, these separated species undergo dehydration polycondensation and gelation reactions.^46^ During the progression of the reaction, there was an observed increase in the concentration of oligomeric gel. This increase leaded to the solidification of heavy metal cations within the system structure, facilitated by the encapsulation effect. Consequently, the desired solidification effect was achieved.^47,48^ The geopolymer's microporous structure and substantial specific surface area facilitate the adequate accommodation of heavy metal ions during the solidification process.

The process of generating hydroxides, carbonates, silicates, and aluminates of heavy metals was occasionally regarded as an alternative method of solidification.^49^ However, it was also recognized as a type of gelation and physical encapsulation, as it relied on the gelation phase for encapsulation and fixation in subsequent stages. Indeed, it was worth noting that heavy metals may not always be exclusively present in the form of ions, as posited by several scholars.^50^ Therefore, Pb^2+^, Cd^2+^, *etc.* can also be immobilized by forming hydroxide precipitate. Hence, it was possible to immobilize Pb^2+^, Cd^2+^, and other similar ions by inducing the formation of hydroxide precipitates.^51^ Additionally, Pb^2+^ ions had the capability to form silicate phases, so enabled their immobilization., and Pb^2+^ could form corresponding silicate phase and been immobilized.^52^ Nevertheless, several experts contended that the presence of the hydroxide phase has not been detected in the infrared spectrum, thus necessitating further experimental evidence to substantiate this mechanism.

Several studies have indicated that the incorporation of pure geopolymer and geopolymer solidified with FA did not introduce additional mineral phases into the existing phase structure. Instead, these materials tended to preserve a significant number of their original features.^53^ This observation suggested that the geopolymer, upon completion of the curing process, did not demonstrate a significant enhancement in its performance with regards to heavy metal ion retention. Nevertheless, it was possible to maintain the original compact structure to a significant degree, hence enhancing its effectiveness in preventing the leaching of heavy metal ions.

Geopolymers had the ability to undergo an alkali activation process involving dissolution, depolymerization, and repolymerization, resulting in the formation of a distinctive three-dimensional network with a zeolite-like cage structure. This structural characteristic enabled geopolymers to effectively solidify heavy metal ions. Furthermore, heavy metals had the potential to contribute to the development of the geopolymer framework structure by engaging in ion replacement processes that arise from electrovalence equilibrium.^54^ The substitution of ions did not alter the inherent fundamental composition of SiO_4_ and AlO_4_. In the composition of the geopolymer, the aluminum ion (Al^3+^) exhibited a notable degree of electronegativity subsequent to its interaction with four oxygen ions (O^2−^). To achieve electrovalence equilibrium, some cations, such as Na^+^ and K^+^, actively engage in the production of the structural monomer, serving to maintain electrovalence balance. The alkali metal ions have the ability to replace certain heavy metal ions present in FA, occupied their positions within the geopolymer structure. This substitution process enabled the stable solidification of these heavy metal ions inside the geopolymer matrix.^55^ The substitution described held particular significance for ions, specifically Pb^2+^, that possessed radii similar to Na^+^ and K^+^. Pb^2+^ has a radius of 0.119 nm, while Na^+^ and K^+^ have radii of 0.095 nm and 0.138 nm, respectively. The proximity of Pb^2+^'s radius to those of Na^+^ and K^+^, coupled with its intermediate position between them, contributed to the notable curative impact of Pb^2+^. This observation further supported the validity of the ion replacement mechanism. Certain studies posited the existence of substituting heavy metals in the covalent structure of Si and Al.^56^ However, it was widely accepted that the primary mechanism of solidification in geopolymers involved the chemical coordination of heavy metal ions with the terminal non-bridge oxygen atoms in Al–O– and Si–O– bonds.

This research aims to create a geopolymer with strong chemical resistance and superior physical characteristics. Geopolymers have shown to be the most promising storage matrix for future industrial applications based on the initial results. Since this work is exploratory, initial results have been achieved, enabling further scientific research to pursue the prospective application of geopolymers for immobilizing heavy mental from FA. Geopolymer have superior mechanical qualities and effective immobilize efficiency. The cured bodies can be utilized in various real-world situations, not limited to landfill sites. Construction, road facilities, mines, and ecological restoration offer a variety of promising opportunities. But the fluctuation in the silica–aluminate content of FA significantly impacts the characteristics of geopolymer.

## 4. Conclusions

This paper studied the safe disposal of FA by geopolymer technology. The effect of oxidize species on the UCS of FA-based geopolymer and the effectiveness of the FA-based geopolymer immobilization of curing heavy metals was explored. Sulfate resistance, resistance to chlorine ion penetration performance and carbonization text were taken to predict the long-term stability of the FA-based geopolymer. The following conclusions can be drawn from this study:

1. The UCS of geopolymer samples was increased with the increase of CaO, and the largest 28 d UCS was 24.8 MPa when CaO content was 31.5%.

2. When the CaO content was 32%, the leaching concentration of heavy metals was the lowest (Pb^2+^ was 0.02 mg L^−1^, Cd^2+^ was 0.01 mg L^−1^), and the S/S rate of heavy metal ions were more than 93.6%.

3. The FA based geopolymer exhibited a high level of resistance to erosion caused by sulfate ions and chloride ions.

4. The results of carbonation tests of FA based geopolymer shown that UCS exhibited a modest rise following the process of carbonation, and then demonstrated a stable trend after a period of 28 days, and the heavy metal leaching test results that comply with the limitations specified in the national standard at 7, 14, 28, and 56 days. The accelerated carbonation ages of geopolymer could be long as 102 years.

5. XRD, FTIR and SEM revived that the three-dimensional structure of zeolites were generated by polymerization reaction in FA-based geopolymer, and solidification mechanisms of heavy metal ions by geopolymer materials could be concluded as gelation, physical encapsulation, and chemical reactions.

## Conflicts of interest

There are no conflicts to declare.

## Supplementary Material

RA-014-D4RA00617H-s001
