# Supplementary material for: Resistance and durability of fly ash based geopolymer for heavy metal immobilization: properties and mechanism
Source: RSC Adv. 2024 Apr 18;14(18):12580–92. doi: 10.1039/d4ra00617h (PMC11025342; doi:10.1039/d4ra00617h)

Electronic Supplementary Material (ESI) for RSC Advances.
This journal is © The Royal Society of Chemistry 2024

Table S1. FA total composition analysis of different regions.

Regions	Cl	SiO_2	Al_2O_3	CaO	Fe_2O_3	MgO	SO_3	Others
1	27.32	3.33	1.00	38.20	0.81	2.00	11.37	-
2	24.52	5.42	1.55	35.26	1.04	1.58	13.27	-
3	29.32	3.30	0.95	32.48	1.22	2.12	15.21	-
4	22.43	3.86	1.37	35.22	0.45	0.97	16.22	-
5	26.32	4.27	1.06	25.24	2.26	3.32	14.25	-

Table S2. FA total composition analysis of different batches.

Batches	Cl	SiO_2	Al_2O_3	CaO	Fe_2O_3	MgO	SO_3	Others
0317	-	13.72	2.38	33.20	0.87	2.10	6.45	-
0330	-	14.60	3.25	40.60	1.63	3.14	6.54	-
0416	-	-	2.69	39.98	1.40	2.45	4.92	-
0422	-	-	1.57	28.50	0.61	1.91	5.46	-
0512	-	-	3.10	34.89	0.94	2.70	5.67	-
0704	-	3.37	1.01	38.20	0.81	2.00	11.37	
0706	29.26	3.33	1.00	38.31	0.73	2.10	10.22	

Table S3. Heavy metal concentration of FA leaching test

Heavy metal	Standard	Detection limit	Measured value
As	0.30	0.0480	0.0303
Ba	25.0	0.0006	1.2311
Be	0.02	0.0001	L
Cd	0.15	0.0040	2.7009
Total Cr	4.50	0.0030	0.0087
Cu	40.0	0.0060	0.9388
Hg	0.05	0.0180	0.0185
Ni	0.50	0.0020	L
Pb	0.25	0.0090	0.3457
Se	0.10	0.0540	0.0212
Zn	100	0.0020	9.4980
Cr^{6+}	1.50	0.0040	0.0050

*L, lower than detection limit

Table S4. Cd and Pb concentration in different batches of FA leaching test

Batches	Cd	Pb
0317	5.78	0.70
0330	0.12	23.6
0416	0.13	11.7
0416	0.02	2.96

0422	0.12	19.3
0422	6.19	0.52
0512	0.12	12.9
0512	1.21	0.07
0520	0.99	0.10
0520	3.22	4.81
0528	0.00	2.83
0528	0.00	3.97
0704	6.12	1.37
0704	10.85	0.62
0706	7.32	1.71
0706	9.78	2.48

Table S5. Evaluation standard of chloride ion impermeability.

6h Electric flux (C)	chloride ion permeability
>4000	High
2000~4000	Mediate
1000~2000	Pore
100~1000	Extremely pore
<100	Negligible

Table S6. Correspondence of accelerated carbonation age and the natural carbonation age

Accelerated carbonation age (d)	7	14	28	56
Natural carbonation age (a)	13	26	51	102

Table S7. Component analysis of solid materials（X-ray Fluorescence Spectrometer）

Chemical composition（%）	CaO	SiO_2	Al_2O_3	Na_2O	K_2O	Fe_2O_3	MgO	others
Metakaolin	1.43	57.61	34.41	2.2	1.63	0.67	0.8	1.25
Slag	36.99	31.53	16.45	0.8	0.71	1.04	7.8	4.68

Table S8. Comparative analysis of measured values of pollutants in ground polymerization curing body and the requirements of standard limits

No.	Project	Cu	Zn	Cd	Pb	Cr	Cr^{+}6	Hg	Ni	As	Fluoride	Dioxin
1	GB5085.3	100	100	1	5	15	5	0.1	5	5	100	—
2	Test value	0.0232	0.0335	0.00012	0.00272	0.00768	ND	0.0007	0.00654	0.0084	0.28	
3	GB5085.6	All compounds limit values in Appendix B require <3% (0.783% measured value for all elements)										≤15000
4	Test value	—	1050	52.2	347	—	—	9.42	10.1	11.1	850	18
5	GB8978	—	—	0.1	1.0	1.5	0.5	0.05	1.0	0.5	—	—
6	Test value	—	—	0.00012	0.00272	0.00768	ND	0.0007	0.00654	0.0084	—	—
7	GB36600	—	—	65	800	—	—	38	900	60	—	40
8	Test value	—	—	52.2	347	—	—	9.42	10.1	11.1	—	18
9	GB14848	1	1	0.005	0.01	—	0.05	0.001	0.02	0.01	1	—
10	Test value	0.0232	0.0335	0.00012	0.00272	—	ND	0.0007	0.00654	0.0084	0.28	—
11	GB3838	1	1	0.005	0.05	—	0.05	0.001	—	0.05	1	—
12	Test value	0.0232	0.0335	0.00012	0.00272	—	ND	0.0007	—	0.0084	0.28	—
13	GB16889	40	100	0.15	0.25	4.5	1.5	0.05	0.5	0.3	—	3000
14	Test value	0.09	0.03	0.04	0.02	0.9	ND	ND	ND	ND	—	18

Remarks: (1) GB5085.3 is 《Hazardous Waste Identification Standards for Leaching Toxicity Identification》 (GB5085.3-2007), GB5085.6 is 《Hazardous Waste Identification Standards for Toxic Substance Content》 (GB5085.6-2007); (2) GB8978 is 《Comprehensive Emission Standards for Sewage》(GB8978-2002); (3) GB36600 is 《Soil Environmental Quality Construction Land Soil Pollution Risk Control Standards》(GB36600-2018); (4) GB14848 is 《Groundwater Quality Standards》 (GB36600-2018); (5) GB14848 is 《Groundwater Quality Standards》 (GB36600-2018); (6) GB14848 is 《Groundwater Quality Standards》(GB36600-2018) GB36600 for 《Soil Environmental Quality Soil Pollution Risk Control Standards for Construction Land》 (GB36600-2018); (4) GB14848 for 《Groundwater Quality Standards》(GB14848-2017); (5) GB3838 for 《Environmental Quality Standards for Surface Waters》(GB 3838-2002); (6) GB16889 for 《Domestic Landfill Pollution Control Standards 》(GB16889-2008). (7) The numerical unit in GB5085.3, GB8978, GB14848, GB3838 and GB16889 is mg/L; the numerical unit in GB5085.6 and GB36600 is mg/kg; and the duality equivalent unit is ng-TEQ/kg.（8）All the above standard documents are from the Ministry of Ecology and Environment of the People's Republic of China（https://www.mee.gov.cn/）

Test S1.

To commence the experiment, extracted a 10g section of the specimen. Proceeded by incorporating the leaching agent in a 20:1 ratio. Subsequently, positioned the sample on the turnover oscillator and setted the rotation speed to 30 ± 2R/min. Allowed the sample to oscillate for a duration of 18 ± 2 hours. The leaching concentration of heavy metals in the FA or geopolymer were assessed using a HJ-E005 atomic absorption spectrophotometer.

$$\varphi = \frac{A_0 - A_1}{A_0} \times 100\% \qquad \text{Eq S1}$$

Where: φ — curing rate of heavy metal ion, %;

Ao — leaching concentration of heavy metal ions in fly ash before solidification treatment, mg/L;

A — Leaching concentration of heavy metal ions in the solidified body after solidification treatment, mg/L.

Test S2.

The solidified body test blocks were prepared by following the specified test ratio, wherein three cylindrical test specimens were prepared for each group. Each test specimen had a diameter of 100 ± 1 mm and a height of 50 ± 2 mm. Following the act of being erected, the test pieces were subsequently transferred to a curing box that maintains a consistent temperature and humidity for a duration of 24 hours. Subsequently, the formwork wass detached. The hardened body obtained following the removal of the formwork was immersed in clean water for a period of 28 days to facilitate the curing process. The employed methodology was the electric flux test method. Initially, the solidified body's test blocks underwent vacuum saturation before tested. The test block was placed within a vacuum container, whereby the vacuum pressure within the container was gradually dropped by 1-5K Pa over a span of 5 minutes. Subsequently, the vacuum level was maintained for a duration of 3 hours. Subsequently,

deionized water was added into the vacuum vessel until the test block was fully immersed. After the restoration of normal pressure, it was recommended to maintain immersion for a duration of 18 ± 2 hours. Once the vacuum water saturation process was completed, the test specimen was removed, any surplus water present on its surface was wiped away, and subsequently, the test specimen was carefully positioned within a mold. Finally, the bolt was securely fastened to affix and seal the test specimen in place. Upon verifying the integrity of the mold, a 0.3 mol/L NaOH solution and a 3% NaCl solution were introduced to the positive and negative electrodes of the power supply, respectively. Following the completion of the inspection, proceeded to activate the power supply and proceed with the task of documenting the values of current A and current C at regular intervaled of 15 minutes. The precise magnitudes of the current and electric flux were precisely 0.01 milliamperes and 0.01 coulombs, respectively.

Test S3.

The detailed process steps were as follows:

a. The dried FA solidified body samples were placed on an iron frame in a carbonization box, and the distance between the carbonized surfaces of each group were at least 50mm;

b. Carbonation test should be carried out in accordance with Standard for Test Methods of Long-term Performance and Durability of Ordinary Concrete (GB/T50082-2009);

c. At 3, 7, 14, and 28 days after carbonization, the samples of the solidified body were taken out and broken to measure the carbonization depth. By comparing the depth of artificial carbonation with that in the reference, the corresponding actual carbonation ages were determined, which were used as a standard to support the corresponding age of the carbonation acceleration test of the FA-based geopolymer;

d. After carbonization for 7, 14, 28 and 56 days, the FA-based geopolymers were taken out and then

sprayed with or dropped with 1% phenolphthalein alcohol solution (containing 20% distilled water). After 30 seconds, the carbonation depth of the solidified body sample should be measured with the carbonation depth measuring ruler according to the original marking of one measuring point every 10 mm.

e. Confirmed the carbonization depth for a certain carbonization time according to the above steps, take three groups of samples respectively, and taken out the FA-based geopolymer with the same carbonization depth to carry out the heavy metal leaching test of the solidified body according to HJ/T 300-2007 and HJ/T 299-2007 respectively to determine the pH value of the leaching solution and the leaching toxicity of heavy metals. At the same time, the FA-based geopolymers were leached with the same amount of water (the same quality as the acetic acid leaching solution), and the pH value and heavy metal leaching toxicity of the leaching solution were tested.

The natural carbonation ages could be calculated by:

$$D_2 = D_1\sqrt{\frac{t_2C_2}{t_1C_1}} \qquad \text{Eq S2}$$

Where, D_2, natural carbonation depth;

D_1, accelerated carbonation depth;

t_1, accelerated carbonation age;

t_2, natural carbonation age

C_1, CO_2 concentration of accelerated carbonation, 20%;

C_2, CO_2 concentration of natural carbonation, 0.03%.

Figures

Figure S1. Soaking processes diagram of geopolymer and test blocks after soaking.

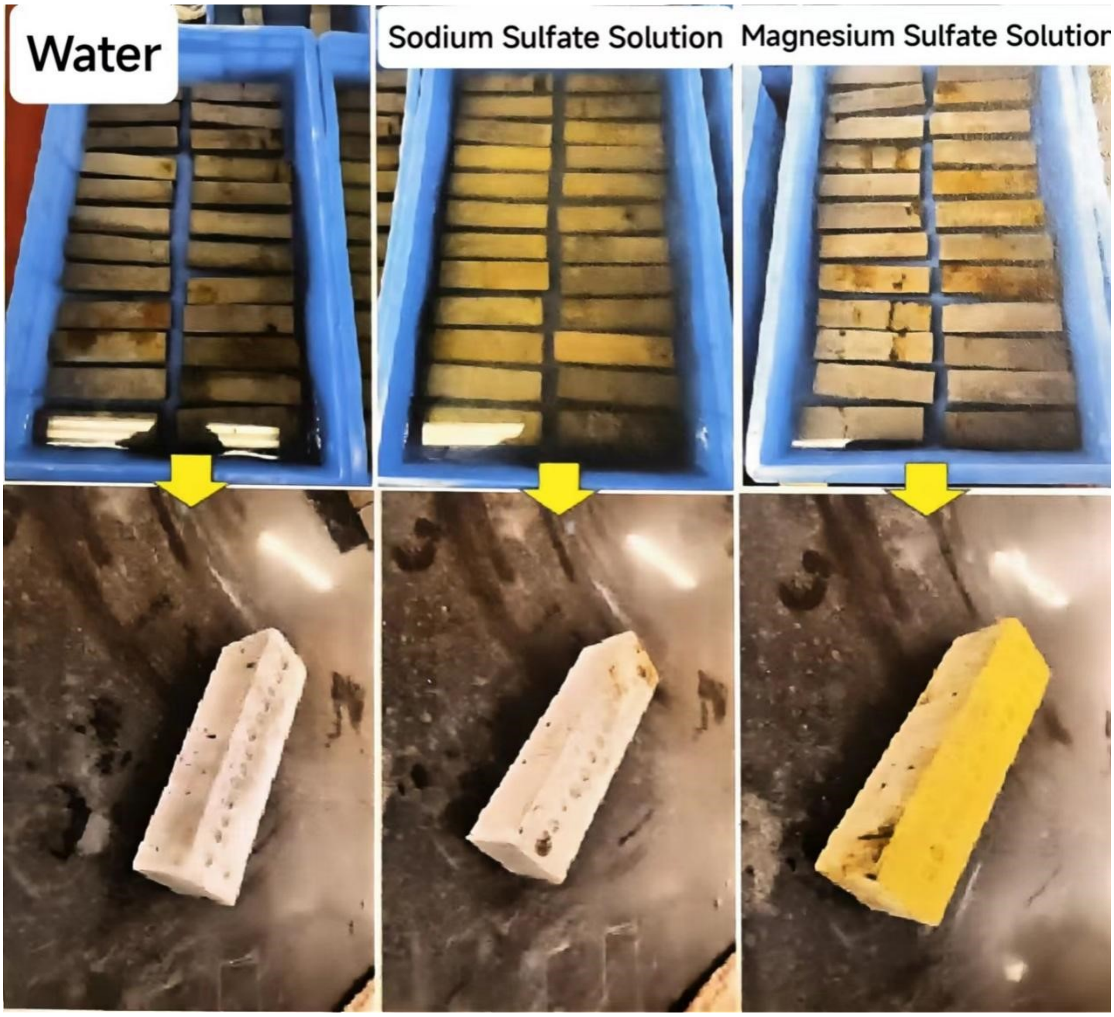

Figure S2. Ddiagram of scheme representing the Synthesis of FA based geopolymer process

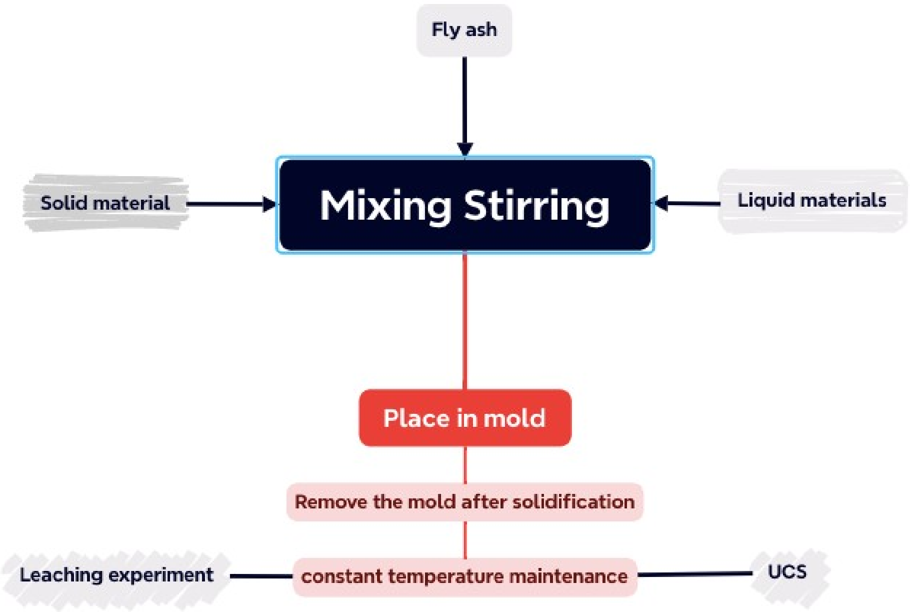

Figure S3. Scanning electron micrograph of raw material before synthesis（a.Metakaolin b.Slag ）

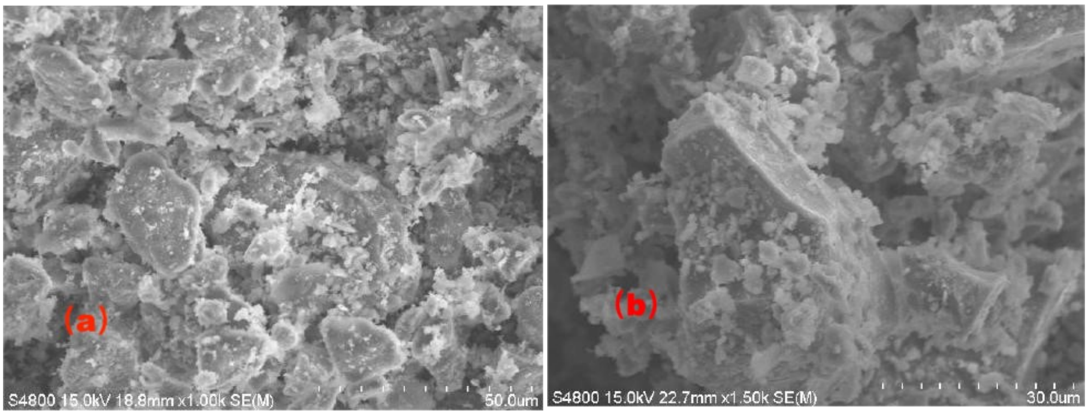

Supplement: RA-014-D4RA00617H-s001 [file RA-014-D4RA00617H-s001.pdf]
